# Establishment of a New Cell Line from Lepidopteran Epidermis and Hormonal Regulation on the Genes

**DOI:** 10.1371/journal.pone.0003127

**Published:** 2008-09-03

**Authors:** Hong-Lian Shao, Wei-Wei Zheng, Peng-Cheng Liu, Qian Wang, Jin-Xing Wang, Xiao-Fan Zhao

**Affiliations:** School of Life Sciences, Shandong University, Jinan, China; Baylor College of Medicine, United States of America

## Abstract

When an insect molts, old cuticle on the outside of the integument is shed by apolysis and a new cuticle is formed under the old one. This process is completed by the epidermal cells which are controlled by 20-hydroxyecdysone (20E) and juvenile hormone. To understand the molecular mechanisms of integument remolding and hormonal regulation on the gene expression, an epidermal cell line from the 5th instar larval integument of *Helicoverpa armigera* was established and named HaEpi. The cell line has been cultured continuously for 82 passages beginning on June 30, 2005 until now. Cell doubling time was 64 h. The chromosomes were granular and the chromosome mode was from 70 to 76. Collagenase I was used to detach the cells from the flask bottom. Non-self pathogen AcMNPV induced the cells to apoptosis. The cell line was proved to be an epidermal cell line based on its unique gene expression pattern. It responded to 20E and the non-steroidal ecdysone agonist RH-2485. Its gene expression could be knocked down using RNA interference. Various genes in the cell line were investigated based on their response to 20E. This new cell line represents a platform for investigating the 20E signaling transduction pathway, the immune response mechanism in lepidopteran epidermis and interactions of the genes.

## Introduction

Insect integument consists of the basement membrane, epidermis, endocuticle, exocuticle, and epicuticle. The epidermis attaching to the basement membrane is a single layer which primarily consists of epidermal cells and a limited number of other cells, such as oenocytes, sensory cells, dermal gland cells, and hair-forming cells. The endocuticle, exocuticle, and epicuticle are excreted by the epidermis and these three layers compose the nonliving cuticle. Because the cuticle is rigid, many molting occurrences are necessary for insect surface area growth from egg to adult. During molting the epidermal cells divide, secrete proteases and chitinases to digest the old endocuticle, absorb the degraded products to produce a new cuticulin (the innermost sublayer of epicuticle), and form a new epicuticle and a new endocuticle outside and inside the new cuticurtin, respectively. The old cuticle is shed once the new cuticle forms. Thus, the epidermis plays a very important role in molting [1].

Molting is conducted by epidermal cells which in turn are controlled by 2 major hormones: steroid 20-hydroxyecdysone (20E) and sesquiterpenoid juvenile hormone (JH) [2]. To understand the hormonal control of the epidermal molecular process during molting, research on molting-related genes has been conducted either *in vivo* epidermis or *in vitro* primary cultured epidermis in *Manduca sexta* [3]. *In vivo* materials, however, cannot evade the undesirable impacts of complicated physiology factors. *In vitro* primary cultured epidermis is inconvenient for large scale experiments. A continual cell line from the epidermis is highly necessary, especially in relation to further study on the hormonal signal transduction pathway.

Many insect cell lines have been established since 1962, including cell lines from *Antheraea pernyi* ovaries [4], the often-used Sf21 [5] and Tn5B1 (HiFive) cell lines from *Spodoptera frugiperda* and *Trichoplusia ni* ovaries, respectively [6], S2 cell lines from *Drosophila* embryos [7], *Spodoptera exigua* fat body cell lines [8], *Helicoverpa armigera* haemocyte cell lines [9], and *Choristoneura fumiferana* midgut cell lines CF-203 [10]. Three epidermal cell lines, including IAL-PID2, from the last instar larval wing disc of Lepidoptera, *T. ni*, *S. frugiperda*, and *Plodia interpunctella*, were established in 1982 and 1983 [11], [12]. Much study has been conducted on hormonally regulated gene expressions with the aid of these insect cell lines [13], [14]. However, until now no epidermal cell line has been determined from the organ-undetermined epidermis of insect larval integument.

Insect molting and metamorphosis depend on the interplay of 20E and JH. Both exist and play important roles during each larval molt, while only ecdyone and 20E govern metamorphosis. When 20E peaks, it binds with its heterodimeric receptors EcR and USP to initiate the early genes, such as the set of transcription factors *EcR*, *USP*, *E74*, *E75*, *BR-C*, *HR3*, and *HR4* [15], and is followed by several relative late genes, including proteinases [16], [17] and other late genes [18], [19].

Three *EcR* isoforms (*EcR-A*, *EcR-B1*, and *EcR-B2*) [20] and one *USP* exist in the higher Diptera, such as *Drosophila melanogaster* [21]. However, 2 isoforms exist in Lepidoptera, *M. sexta*: *EcR* (*EcR-B1* and *EcR-A*) [22], [23] and *USP* (*USP-1* and *USP-2*) [24]. The *E75* isoform varies widely. *E75A* and *E75B* have been previously cloned [25], whereas 2 other isoforms of *E75* (*E75C* and *E75D*) were only obtained recently [26]. Most of these genes are regulated by ecdysteroid [27]. For instance, when the ecdysteroid titer of the 4th larval instar tobacco hornworm begins to increase, the mRNAs of both *EcR-B1* and *E75A* increase and then decline gradually just as ecdysteroid peaks. Another notable transcription factor is *MHR3*. MHR3 mRNA is not expressed when ecdysteroid titer levels are low; however, it appears just before head capsule slippage (HCS) [28]. Recently, Siaussat *et al.* investigated the functions of *EcR*, *USP*, and *HR3* in the 20E signal pathway in the IAL-PID2 cell line [29]. All of these genes represent good targets for investigating the hormonal signal transduction in gene expression.

Here we report the epidermal cell line HaEpi that was established and derived from the integument epidermis of the 5th-molting *Helicoverpa armigera* larvae. Ten of the genes from upstream to downstream involved in the hormonal signal pathway during molting were subsequently investigated. This cell line not only represents a platform for investigating hormonal regulation on the gene expression in the epidermal cell line, but also represents a model for: (1) investigating the apoptosis mechanism that occurs during virus invasion, and (2) performing RNAi to investigate gene function.

## Results

### Establishing the new cell line from epidermis

In the primary culture, a small number of round and bright cells appeared around a piece of epidermal tissue 30 days after epidermal tissue pieces were inoculated. These cells increased in number and grew denser 40 days post-inoculation and were gently dispersed with a pipette. One week later, cells attached and spread to form discrete patches on the flask bottom. During later subculture, cells adhered tightly to the flask bottom and were not sensitive to 0.25% trypsin containing 0.02% EDTA but sensitive to collagenase I. Cells were digested with 0.1% collagenase I for approximately 20 min and separated from the flask bottom. Since its establishment, the cell line has been subcultured for more than 82 passages and was named HaEpi.

**Figure 1:**
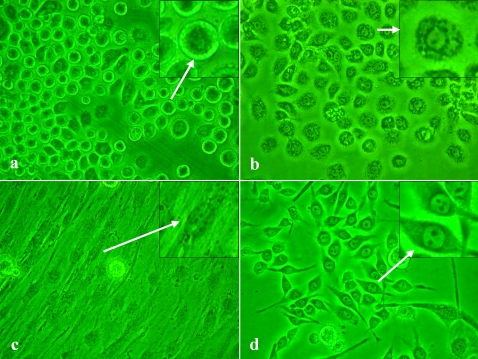
Morphology of the HaEpi cell line established from larval epidermis of *H. armigera* subcultured 12th passage. Observed under phase contrast microscope (10×40). Panels a and b, two kinds of epithelial cells; c, fibroblast-like cell; d, spindle-like cell.

**Figure 2:**
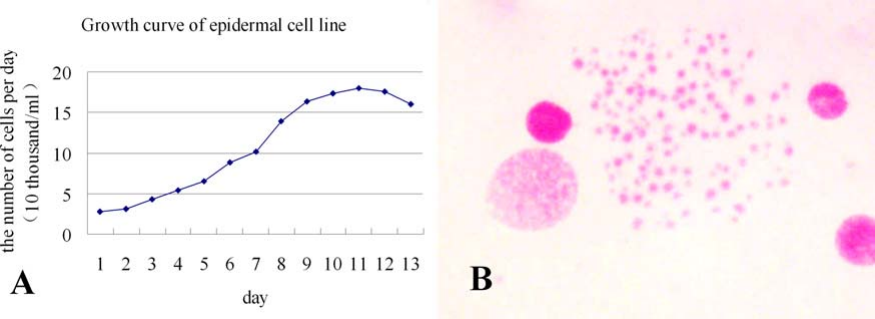
Analysis of the growth curve and chromosomes of the cell line. A, growth curve of epidermal cell line HaEpi in Grace's medium containing 10% fetal bovine serum at 27°C. B, chromosomes of the HaEpi cell line. 10×100.

Early passages resulted in the observation of 4 cell types. Epithelial cells formed the primary cell type (70%), and included 2 shapes: (1) rounding with unclear nuclear (Fig. 1A), and (2) stretching with clear nuclear (Fig. 1B). Fibroblast-like cells (Fig. 1C, 1.9%) and spindle-like cells (Fig. 1D, 28%) were also observed. Fibroblast-like cells in Fig. C gradually disappeared over the 10 passages. Only epithelial cells in Fig. A, B and spindle-like cells in Fig. D survived for later experiments (Fig. 1).

### Growth curve and cell doubling time

Growth curve was measured at the 12th passage. The curve demonstrates that the logarithmic growth phase occurred 5 to 10 days after subculturing. The saturated phase occurred on day 11, after which cells aged and died. Saturated cells formed approximately 3.6×10^5^ cells in a 35-mm well, which denoted a 6.5-fold increase over the initial population (5.5×10^4^ cells per well) after 10 days. Population doubling time during logarithmic growth was calculated at 64 h using the Hayflick formula (Fig. 2A). Each mitosis cell contained numerous granular-shaped chromosomes. Chromosome numbers were counted randomly under a microscope using 100 samples. The result revealed that this cell line demonstrated great variation in chromosome numbers (30 to 820) and the chromosome mode was 70 to 76 (Fig. 2B).

### Virus susceptibility of the cell line

**Figure 3:**
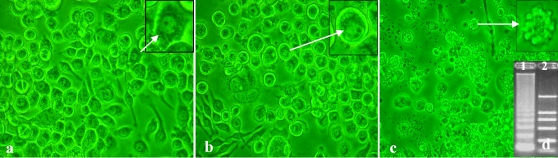
Cytopathology character of HaEpi cells infected with baculovirus. Panel a, uninfected HaEpi cells; panel b, HaEpi cells infected with HaSNPV; panel c, HaEpi cells infected with AcMNPV; panel d, DNA ladder of apoptotic HaEpi cells induced by AcMNPV, 1, DNA ladder of apoptotic HaEpi cells, 2, DL2000 DNA marker. Arrow indicates normal cell in a and b, and apoptotic cell in c. Observed 4 days after virus infection. 10×40 magnified.

Cytopathology characteristics of cells infected with 2 kinds of baculovirus were observed. Morphology of the epidermal cells infected with HaSNPV was almost similar to that of the non-infected control cells except for the fact that very few cells were infected with HaSNPV and occluding bodies produced in nuclei (Fig. 3A, B). However, apoptosis occurred in the epidermal cells after 24 h post-infection (hpi) with *Autographa Californica* multiple nucleocapsid nucleopolyhedrovirus (AcMNPV) (Fig. 3C). The highest ratio of apoptosis was approximately 70%. Total DNA of the cells 72 hpi with AcMNPV revealed a “DNA ladder” under agarose electrophoresis (Fig. 3D).

### Identification of HaEpi cell line

**Figure 4:**
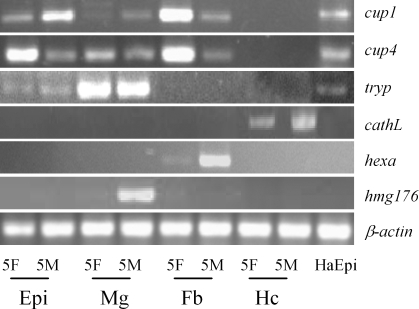
Semi-quantitative RT-PCR to compare gene expression patterns in the HaEpi cell line and tissues. 5F, feeding 5th instar larvae; 5M, molting 5th instar larvae; HaEpi, HaEpi cell line; Epi, epidermis; Mg, midgut; Fb, fat body; Hc, haemocyte.

Expression patterns of some genes were analyzed to identify the epidermal cell line, including 2 cuticle proteins (*Ha-cup1* and *Ha-cup4*), *Ha-trypsin2*, cathepsin L (*Ha-catheL*), hexamerin and *hmg176*. Results showed that two cuticle proteins (*Ha-cup1* and *Ha-cup4*) and *Ha-trypsin2* expressed in both HaEpi cell line and epidermis but did not express in haemocytes. In contrast, *Ha-catheL* expressed only in haemocytes and not in other tissues or the HaEpi cell line, Although *Ha-cup1*, *Ha-cup4* and *Ha-trypsin2* also expressed in the midgut or fat body, *hmg176* expressed only in the midgut and hexamerin only expressed in the fat body but not in HaEpi cell line and epidermis. These facts indicated that HaEpi cell line was not derived from haemocytes, midgut or fat body (Fig. 4).

### Inducibility and suppressibility of gene expression in the cell line

**Figure 5:**
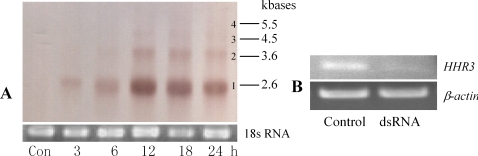
Induction and knock down of *HHR3* expression in HaEpi cell line. A, Northern blot to show induced expression of *HHR3* by RH-2485. Con, control was equal volume of isopropanal. 18 s rRNA was used as a qualitative and quantitative control for RNA. 10 µg total RNA, 1% gel. 3, 6, 12, 18 and 24 are periods (hour) after induction. B, RT-PCR to show knock down of *HHR3* by RNAi, 12 h induction by 20E.

To test the response of the HaEpi cell line to the 20E, we used the non-steroidal ecdysone agonist RH-2485 to induce the cell line and examined the expression of the *Helicoverpa* hormone receptor 3 (*HHR3*). Results showed that *HHR3* was upregulated after being induced by RH-2485. A Dig-labeled *HHR3* probe was used to detect 4 isoforms of *HHR3* transcripts. Band 1 was dominant and could be induced within 3 h, peaked at 12 h, and declined thereafter. The other bands were notably fainter. This result indicated that the cell line responded well to the 20E analogs, which suggested that a new epidermal cell line which could be induced by ecdysone was successfully developed (Fig. 5A). Additionally, *HHR3* expression could be knocked down using the RNAi method. These characters present us with a model for investigating gene function by RNAi in the cell line (Fig. 5B).

### Hormonal regulation on the genes

**Figure 6:**
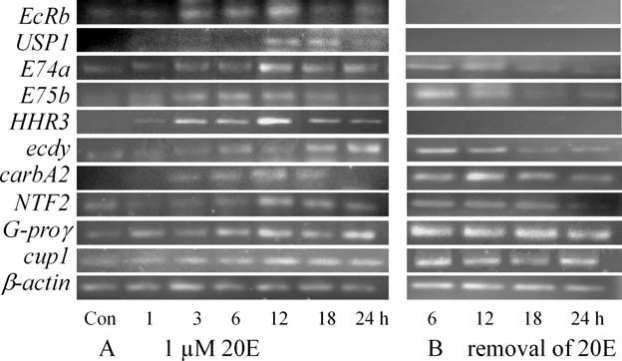
Hormonal regulation on the genes in HaEpi cell line. A, induced expression of the genes in the cells with 1 µM 20E for various hours; B, gene expression patterns after removal of and post 12 h incubation in 20E. Con, control with equal volume of DMSO.

To investigate hormonal regulation on the molting related genes, we performed serial experiments on the HaEpi cell line. Genes involved in the molting cascade from upstream to downstream were examined following hormonal induction. During the 20E induction, nearly all of the examined genes were upregulated by 20E treatment. Ecdysone receptor (*EcRb*) expression peaked at 3∼12 h, while ultraspiracle protein (*USP1*) appeared obviously after 12 h induction with 20E. Ecdysone induced protein E74 (*E74a*) and E75 (*E75b*) increased along with the culture over 24 h. Hormone receptor 3 (*HHR3*) was not detected in the absence of 20E, but rapidly elevated after culturing for 3 h with 20E, peaked at 12 h, and declined gradually thereafter. The ecdysteroid-regulated gene (*ecdy*) increased after induction with 20E. Carboxypeptidase (*carbA2*) expression was similar to *ecdy*. Notably, nuclear transfer factor 2 (*NTF2*) and G protein *γ* subunit (*G-proγ*) were also upregulated by 20E (Fig. 6A). Otherwise, withdrawing of 20E after 12 h culture in it, *EcRb*, *USP1* and *HHR3* stopped expression at 6 h. Other genes also showed decreased expression along with the incubation time from 6 to 24 h after withdrawal of 20E. The cuticle protein 1 (*cup1*) did not demonstrate a close relationship to the withdrawing of 20E (Fig. 6B).

## Discussion

A new epidermis cell line, HaEpi, was established from the penultimate (5th) instar larval integument of *H. armigera*. HaEpi comes from undifferentiated and undetermined epidermis. This cell line differs from the 3 epidermal cell lines from the imaginal wing discs of last instar larvae developed by Lynn *et al.*, which are undifferentiated but determined primordia assembled into organized packets of cells in immature holometabolous insects such as Diptera and Lepidoptera [11], [12]. Therefore, the HaEpi cell line may reveal different characteristics upon further study.

HaEpi also differs from Sf21. The HaEpi epidermis cells adhered to the bottom of flask very tightly and could not be easily separated from the culture flask bottom either by the mechanical method or when digested by trypsin (0.125∼0.5%, or mixed with 0.02% EDTA). In contrast, the epidermis cells were easily separated using collagenase I. Animal epidermal cells often synthesize collagens and excrete them to an extracellular matrix to produce high tensile strength, which allows cells to adhere tightly to the basement membrane or results in tissue fastness. The fact that the HaEpi cell line can be separated by collagenase I suggests that the cells synthesize and excrete collagen and retain the characteristics of the integument cells from which they came. This does not exist in any other previously established insect cell line.

The unique gene expression pattern in the HaEpi cell line indicates that this is an epidermal cell line. We previously reported that *Ha-cathL* was expressed only in haemocytes [30]. So we used *Ha-cathL* to exclude the possibility of haemocyte contamination in the HaEpi cell line. The fact that *Ha-cathL* expressed only in haemocytes but not in the HaEpi cell line confirmed that the newly-established cell line was not derived from haemocytes. Additionally, the fact that *Ha-cup1*, *Ha-cup4* and *Ha-trypsin2* did not express in the haemocytes but expressed in the epidermis and the new HaEpi cell line also confirms the above-noted conclusion. Although *Ha-cup1*, *Ha-cup4* and *Ha-trypsin2* expressed in the midgut or fat body in addition to the epidermis and HaEpi cell line, the midgut and fat body cannot contaminate the cell line because they were excluded by dissection upon primary preparation of the epidermal tissue. Furthermore, the expression patterns of *hexamerin* and *hmg176* confirmed that fat body and midgut cannot contaminate the cell line. Therefore, we obtained an epidermal cell line from the *Helicoverpa* larval epidermis. The reason that *Ha-cup1 and Ha-cup4* expressed in the midgut and fat body in addition to the epidermis might be due to the trachea distribution in these organs, which also contains epithelium cells inside its inner cavity, or may be due to the fact that these organs also express cuticle proteins.

The induction expression of *HHR3* by non-steroidal ecdysone agonist RH-2485 indicated that the HaEpi cell line responds to the ecdysone hormone well. *Helicoverpa armigera* Hormone receptor 3 (*HHR3*), a molecular indicator of insect molting, was a transcription factor involved in molting [31]. RH-2485 was used to investigate the 20E response of the *HHR3 in vitro* or *in vivo* [32]. Thus, the inducibility of *HHR3* by RH-2485 suggested that the HaEpi cell line is able to respond to 20E and can be used to investigate the 20E signal transduction pathway. Similarly, knock down of *HHR3* via RNAi indicated that this cell line can be used to investigate gene function in various signaling pathways by RNAi technique.

Further experiments on hormonal regulation of gene expression revealed that the HaEpi cell line is not only a good platform for 20E hormone regulation on genes, but also revealed expression patterns of the molting related genes under regulation of hormones. It was known that *EcR*, *USP*, *E74*, *E75*, and *HR3* are molting transcriptional factors, which express earlier during the molting cascade [28]. We have also reported that *ecdy*, *carbA2*, *NTF2*, and *G-proγ* are upregulated in molting larvae or metamorphic committed larvae, which might be the late genes involved in the molting process because they are effector genes [30]. The expressions of these genes rely on the existence of 20E in the HaEpi cell line, which reveals the hormonal regulation patterns on these genes.

The fact that the non-self pathogen AcMNPV induced the cell line to apoptosis represents a good model for studying the immune response signal pathway. Additionally, the numbers of chromosomes of the *in vitro* cultured epidermis cells varied from 30 to 820. Vast numbers of chromosomes is very common in insect cells. Epidermal cells of the final instar *Manduca* caterpillar range in chromatin ploidy from 40 to 640. Polyploidy are possibly associated with a cell's ability to synthesize large amounts of protein [33].

In sum, we established a new insect epidermis cell line from the undetermined epidermis of the 5th instar larval integument of *H. armigera*. Non-self pathogen AcMNPV induced apoptosis to occur in the cell line. The cell line is able to respond to 20E and RH-2485, and its gene expression is suppressible by RNA interference. This presents a new cell line platform or model for investigating the hormonal and other signaling transduction pathways in lepidopteran epidermis.

## Materials and Methods

### Chemicals

Grace's insect cell culture medium and yeastolate was obtained from Gibco (Grand Island, New Jersey, USA; Paisley, Scotland, UK), fetal bovine serum was obtained from MDgenics Incoperated (St.Louis, Missouri, USA), Biozol reagent was obtained from Bori Company (Hangzhou, China), and M-MLV reverse transcriptase was obtained from Invitrogen (Carlsbad, California, USA). 20-hydroxyecdysone was obtained from Sigma (St. Louis, Missouri, USA; Steinheim Germany). Collagenase I was obtained from Worthington Biochemical Corporation (Lakewood, New Jersey, USA). RH-2485 (95% pure) was donated by the Rohm and Haas Company (Spring House, Pennsylvania, USA). Lactalbumin hydrolysate was obtained from Merck (Whitehouse Station, New Jersey, USA). PCR purification kit (Shanghai Shengong Biological Engineering Technology & Services Co. Ltd, Shanghai, China). MEGAscript™ RNAi kit (Ambion Inc. Ausdin, USA).

### Insect

Eggs from the cotton bollworm *H. armigera* were sterilized with Formalin (5%) for approximately 10 min. Larvae were reared on an artificial diet at 28°C under a daily photoperiod of 16 h and 60% humidity. Moths were fed 2% sugar water. The artificial diet for *H. armigera* is described in [34].

### Continual culture and preservation of the epidermis cells

The tissue mass cell culture method was used in this study. Fifth instar larvae in the head capsule slippage stage were immersed in scrap ice until they became faint. Larvae were washed twice to remove surface contamination and sterilized by submersion in 70% alcohol for approximately 3 min. Sterilized larvae were placed in a sterilized tissue culture dish. Dorsal integuments were severed carefully with sterilized scissors and washed with culture medium to remove haemolymph. The fat body, tracheae, and muscle adherent to the epidermis were carefully removed. Epidermis was then scraped from the cuticle with the obtuse end of tweezers and transferred to a 3.5 cm culture plate. Epidermis tissue was cut into small pieces with sterilized scissors and explanted into a new flask. Tissue masses were dispersed and incubated at 27°C after being dripped into 0.5 ml primary culture medium (Grace's Complete Insect Medium supplemented with 20% fetal bovine serum and 50 IU gentamicin/ml). The following day, 1.5 ml primary culture medium was added. After 10 days, 1.5 ml of medium was added. Cells in the flask were examined under a phase contrast microscope every 2 or 3 days. Then, half of the medium in the flask was replaced with fresh medium every 10 to 15 days according to the growth state of the cells.

Mechanical disintegration or the digestive method was used to subculture cells. When cultured cells grew to a confluent layer, subculture was conducted. The first subculture method used was as follows: (1) cells were gently rinsed away from the monolayer with culture medium using a sterilized pipette or by tapping the bottom of the flask to release attached cells; (2) the cell suspension was transferred into a new 25 cm^2^ flask at a ratio of 1∶2 and were supplied with an equal volume of fresh medium. After cells were successfully subcultured for several times, another subculture method was used as follows: (1) cells were separated via digestion with collagenase I which dissolved in D-Hanks' balanced salt solution (D-HBSS, NaCl 136.75 mM, NaH_2_PO_4_·H_2_O 0.36 mM, KCl 2.68 mM, NaHCO_3_ 11.90 mM, sodium citrate C_6_H_5_Na_3_O_7_·H_2_O 3.62 mM, D-glucose 5.62 mM) with 2 mM CaCl_2_, and (2) cells were subsequently transferred into a new 25 cm^2^ flask at a ratio of 1∶2 and were supplied with an equal volume of fresh medium. During the 5th-10th passages, fetal bovine serum in the culture medium was gradually reduced from 20% to 10%.

Cells were frozen from the 5th passage. The freezing medium comprised 90% primary culture medium (20% FBS) and 10% dimethyl sulfoxide (DMSO). When cells grew to 80∼90% confluences, approximately 3×10^6^ viable cells from the tissue culture flasks were collected. The cell suspension was centrifuged at 200 g for 5 min. The cell pellet was resuspended in the pre-determined volume of chilled freezing medium, mixed to a homogeneous cell suspension, and transferred into a freezing tube. Cells were manually frozen to −20°C at a rate of decrease of 1°C per min and transferred to liquid nitrogen storage.

The cell line thawing procedure was as follows: (1) the vial of frozen cells was removed from liquid nitrogen and thawed quickly in a 37°C water bath; (2) cells were transferred to a sterile 15 ml tube containing primary culture medium; (3) cells were centrifuged briefly at 200 g, washed again, and then resuspended in 2 ml primary culture medium; (3) cells were transferred to a 25 cm^2^ flask containing 5 ml of complete medium; (4) the flask was incubated at 27°C to allow the cells to attach to the bottom of the flask; and (5) the following day, the medium was aspirated and replaced with fresh medium.

### Growth curve of the cells

Cells were inoculated in seven 6-well plates at 3×10^4^ cells/ml (well cell suspension = 2 ml). From the following day, cell numbers were counted using a haemocytometer. Counting occurred in succession for 14 days and 3 wells every day. The growth curve was generated such that horizontal coordinate datums defined days and vertical ordinate datums defined cell densities. Cell doubling times during logarithmic growth were calculated according to Hayflick's formula T = t·lg2/lg(N/N_0_) (T = population doubling time; t = appointed time after subculture; N = number of cells at the appointed time; N_0_ = number of cells at the beginning of subculture) [35].

### Chromosome analysis of the cells

Chromosomes were prepared at the 18th passage. When cells in a 25 cm^2^ flask grew to the log-phase, colchicine was added in the medium at a final concentration of 0.1 µg/ml. After 15 h, cells were collected and centrifuged at 1000 rpm for 10 min. The pellet was washed with PBS (33 mM KH_2_PO_4_, 33 mM Na_2_HPO_4_, pH 6.8) and then resuspended in 4 ml hypotonic solution of 0.5% KCl for 10 min at 27°C, after which, 4 ml fixatives (methanol∶glacial acetic acid 3∶1) were added into cells for 5 min. Cells were gathered and fixed in 4 ml fresh fixatives for 20 min. Fixed cells were centrifuged and resuspended in 0.5 ml fixatives and dropped vertically onto cold slides. After air drying, the slide was stained with Giemsa for 20 min and chromosomes were observed and counted under a light microscope. Chromosomes and cell nuclei were stained mauve with Giemsa.

### Virus susceptibility

Susceptibility of the cell line to HaSNPV or AcMNPV was tested. First, budded virus (BV) was prepared. The third or fourth instar larvae of *H. armigera* (average weight 0.004 g/larva) were starved for 16 h and inoculated orally with occluding body (OB) viruses (1×10^7^) confected by blue eatable dye using the droplet feeding method. Three days later, the larval haemolymph with BV was harvested from the larval abdomen foot, diluted at 1∶10 with culture medium, and filtrated. 1×10^6^ cells were seeded into a 25 cm^2^ flask. When cells grew and arrived at the logarithmic growth phase, 1.5 ml medium containing BV of HaSNPV or AcMNPV was inoculated in the flask and incubated for 1.5 h. The medium containing BV was replaced with fresh medium and cells were cultured continuously. Cells were observed under a phase contrast microscope daily post-infection until OV appeared or apoptosis occurred. DNA from the cells 4 days post-infection was extracted and underwent agarose gel electrophoresis.

### Northern blot analysis of HHR3

The entire experiment was performed following the procedure provided by Roche (Boehringer Mannheim, Mannheim, Germany). 10 µg total RNA isolated from RH-2485-treated cells or cells incubated with an equal volume of isopropanol were used in the analysis. After electrophoresis on an agarose gel, RNA was transferred to a nylon membrane followed by a UV crosslink for 10 min. After prehybridizing for 2 h at 68°C, blots were hybridized with the digoxigenin-labeled probe overnight at the same temperature. The membrane was washed with 2×SSC+0.1% SDS for 2×5 min at room temperature, then washed with 0.1×SSC+0.1% SDS for 2×15 min at 68°C. After incubation with Anti-Dig-phosphatase AB for 1 h, the induced expression pattern of the HHR3 transcription factor by RH-2485 was detected using 5-bromo-4-chloro-3-indolyl phosphate (BCIP) and nitroblue tetrazolium chloride (NBT). The digoxigenin labeled antisense HHR3 RNA probe was the same as that used in our previous work [31].

### RNAi

The primers of HHR3RNAiF 5′-gcgtaatacgactcactataggaagggtttcttcaggcgatc-3′ and HHR3RNAiR, 5′-gcgtaatacgactcactatagggttggtatttgcgtgtgcttc-3′ were used for PCR to amplify the gene fragment (545 bp). PCR product purified using a PCR purification kit was used to synthesize dsRNA *in vitro* using the MEGAscriptTM RNAi kit. dsRNA purity and integrity were determined using agarose gel electrophoresis. dsRNA was isopropanol precipitated and suspended in RNase-free water. The cells were cultured at 26°C with Grace's medium supplemented with 10% FBS to 80% confluence and then changed to an FBS-free medium. 1 ml mix of FBS-free medium that contained 8 µg dsRNA and 10 µl Lipofectamine 2000 was diluted to 4 ml by Grace's medium and directly added to cells. The final concentration of dsRNA was 2 µg/ml in the FBS-free medium. After incubation at 26°C for 12 h, cells were rinsed and then re-fed with a normal medium containing 20E at 0.4 µM. After 12 h of culturing, total RNA was isolated from cells for RT-PCR analysis. Control cells were prepared using the same amount of Lipofectamine 2000.

### Hormonal regulation of genes

Epidermal cells at log-phase were cultured for 12 h in a subculture medium containing 20E or RH-2485, which first dissolved to 1 mM/ml in DMSO (isopropanol for RH-2485) and then diluted to a medium of 1∶1000. Control cells were treated with an equal volume of DMSO or isopropanol. To detect gene transcription, total RNAs were extracted from treated cells using Biozol reagent and quantified spectrophotometrically at 260 nm. 2 µg total RNA was reverse transcribed into single-stranded cDNA using M-MLV Reverse Transcriptase.

### Semi-quantitative RT-PCR

Semi-quantitative RT-PCR was performed with primers of various genes (ecdysone receptor B, *EcRB*, EcRBF2 5′-aattgcccgtcagtacga-3′/EcRBR3 5′-tgagcttctcattgagga-3′; ultraspiracle protein, *USP1*, USPF3 5′-ggtcctgacagcaatgtt-3′/USPR3 5′-agctccagctgactgaag-3′; *E74a*, E74F2 5′-tgcaggaccgcgagtact-3′/E74R2 5′-gctggtagtagtagcgca-3′; *E75b*, E75F3 5′-cgccaactgattctggcat-3′/E75R3 5′-acaggcatgtcgtcggct-3′; Ha-hormone receptor 3, *hhr3*, HHR3F2 5′-aagggtttcttcaggcgatc-3′/HHR3R2 5′-gttggtatttgcgtgtgcttc-3′; carboxypeptidase A2, *carbA2*, CarbA2F2 5′-ggaagcagtatcatagactgg-3′/CarbA2R2 5′-atcttgaagctcctttgcctc-3′; ecdysteroid-regulated gene, *ecdyr*, EcdyrF 5′-ctgcaggatgccgtcatac-3′/EcdyrR 5′-ctaggcgcgcggaggagcgat-3′; nuclear transfer factor 2, *NTF2*, NTF2F 5′-atggcgctcaatccacaatac-3′/NTF2R 5′-ctttgaaggagtacagttgca-3′; G-protein-γ, *gproγ*, GproγF 5′-atggatatgatggtatcaacg-3′/GproγR 5′-ttaaagaacagtgcaggaact-3′; cuticle protein 1, *cup1*, Cup1F 5′-atgaaatccttcatcgtactc-3′/Cup1R 5′-ttaggcagccacggggaggtg-3′; cuticle protein 4, *cup4*, Ha-cup4F: 5′-atgaaattcatcatcgttgtc-3′ and Ha-cup4R: 5′-ttatctcctggcggggacc-3′; Ha-trypsin2, *tryp*, Tryp2F2: 5′-tgattggcttgcttgtgcg-3′ and Tryp2R2: 5′-ctcaggaaaagctctaacag-3′; Ha-cathepsin L, *Ha-cathL*, Ha-cathLF 5′-gcggaatacggacaatacac-3′ and Ha-cathLR 5′-gttcagatcgttaggaag -3′; hexamerin, *hexa*, HaHexaF: 5-aggagcaacctctcgcagaaag-3′ and HaHexaR: 5′-tgacgggaagacttcaggaag-3′; *hmg176*, hmg176F: 5′-atgaaaagtttccttgtcatc-3′ and hmg176R: 5′-ttaatctaaccaagaaaccacc-3′; beta-actin, *β-actin*, ActinF 5′-agtagccgccctggttgtagac-3′/ActinR 5′-ttctccatgtcgtcccagt-3′). PCR procedure was as follows: 94°C for 3 min, followed by 28 cycles of 94°C for 30 s, 53°C for 45 s, 72°C for 20 s, and 72°C for 10 min. The *β*-actin gene was used for normalization. The amplification product was then detected in 1 or 2% agar gel.
