# Correction: Establishment of a New Cell Line from Lepidopteran Epidermis and Hormonal Regulation on the Genes

**DOI:** 10.1371/annotation/5957bf19-a800-485d-aa5b-dfcdd095ca13

**Published:** 2008-10-06

**Authors:** Hong-Lian Shao, Wei-Wei Zheng, Peng-Cheng Liu, Qian Wang, Jin-Xing Wang, Xiao-Fan Zhao

The first two authors, Hong-Lian Shao and Wei-Wei Zheng, should be noted as having contributed equally to this work.

